# Intelligence

**Published:** 1808-08

**Authors:** 


### INTELLIGENCE.

M. Perperes, apothecary at Azilles, has published some curious experiments, for the purpose of ascertaining the nature of the acid formed in indigestion. From these he deduces the following results: 1. That the distention of the stomach in cases of indigestion, is occasioned by the formation of carbonic acid, arising from a commencement of decomposition, which the nutritive substances taken as food, chiefly when they are of the mucilaginous vegetable kind, have undergone. 2. That the burning pain which the digestive organ experiences, and which sometimes extends to the œsophagus, is owing to a quantity of acetous acid, formed by the complete disoxygenation of the aliment. 3. That eight ounces of roasted chesnuts produced two ounces and six drachms of acetous acid; after having fermented in the stomach an hour and a half. 4. That the method (which has at least constantly succeeded with him) for remedying this disagreeable sensation, which frequently occurs to persons who have weak stomachs, is to take after a meal ten grains of powdered colombo-root, with twelve grains of calcined magnesia, mixed together for a single dose.

Mr. Accum having recently analysed the water of the different springs at Cheltenham, gives the following particulars respecting the chalybeate strong saline well. The water taken fresh from the pump has a distinct saline taste with a slight impression of bitter. It is colourless, perfectly transparent, without smell, and possesses a strong refractive power. Its temperature was 55° at 295 barometrical pressure, the temperature of the air being 65° Fahr. Its specific gravity was as 2,039 to 2,066. On pouring the water at the fountain-head from one vessel into another, and leaving it exposed to the air, it emits a multitude of exceedingly minute air-bubbles, which firmly adhere to the inner surface of the vessel. Exposed to the open air for eight days, it suffered no material change. This spring yields upwards of 800 gallons of water in 24 hours in every season of the year.

A letter transmitted to the Navy Board, by Captain Hodgson, of his Majesty's Ship Trusty states, that the apparatus of Mr. Lamb, patentee of an invention for distilling fresh water from sea water, used in the above vessel, performed very well, and consumed less fuel than one before in use for the same purpose; that the operation of distillation does not in the least interfere with the cooking of the ship's company's meat; that when three boilers are in use, from twenty to twenty-five gallons of fresh water per hour are produced; and that though the water at first is not perfectly agreeable to the taste, though clear, yet when exposed to the air for a short time, it becomes very good. The apparatus for the Trusty was of the size calculated for a fifty gun ship.

The Society for the Relief of the Ruptured Poor, upon a Plan some time ago recommended to the Public, through the medium of several public Journals, has at length assumed a regular form. The

Right Honourable the Lord Mayor, for the time being; is President; and the Committee for managing the affairs of this Charity, consists of twenty-four Governors, among whom are some of the first Medical Characters in the metropolis. The Governors of the City Dispensary have generously permitted the affairs of the above Institution to be conducted at their establishment in Grocer's Hall Court, Poultry; and the surgical and other officers accept of no gratuity whatever for their services; by this laudable economy the whole of the funds being exclusively devoted to the purposes of the Charity.—Each Contributor of a Guinea annually, will have a right to recommend three patients during the year, each of whom will receive a Truss, besides medical and surgical attendance. Subscriptions will be received, and Plans of the Charity may be obtained, upon application to James Amos, Esq. Devonshire Square, Bishopsgate Street, Treasurer; John Taunton, Esq. Greville Street, Hatton Garden, Surgeon to the Institution; Mr. Bartlett, at the Finsbury Dispensary; Mr. Elliott, at the City Dispensary; and Mr. A. B. Turnbull, Bolt Court, Fleet Street, Secretary.

Dr. Clough, Physician and Manmidwife to the St. Mary-le-bone General Dispensary, &c. will on Monday the 8th of August, 1808, at ten in the morning, commence his Course of Lectures on Puerperal Anatomy, Physiology, and Pathology. A Syllabus, and Prospectus of which may be had of Dr. Clough, No. 68, Berners Street; J. Callow, Crown Court, Soho; and at the Dispensary, No. 77, Welbeck Street, Cavendish Square.—An Evening Course will also be given for the convenience of Students attending other Classes in the forenoon, on Tuesdays, Thursdays, and Saturdays, at seven; the first Lecture commencing on the evening of the 9th instant.

In two or three months, Mr. Watt of Paisley, will publish a work on Diabetes, consisting chiefly of "Cases and Observations." The practice Mr. W. has adopted is new, and very different from that which has been generally followed of late years.—There will also be subjoined, "Cases alluding to Diabetes," where a similar treatment has been successful.

